# Lack of relationship between EGFR-1 immunohistochemical expression and prognosis in a multicentre clinical trial of 93 patients with advanced primary ovarian epithelial cancer (*GINECO* group)

**DOI:** 10.1038/sj.bjc.6601961

**Published:** 2004-06-29

**Authors:** C Elie, J F Geay, M Morcos, A Le Tourneau, V Girre, P Broët, B Marmey, L Chauvenet, J Audouin, E Pujade-Lauraine, S Camilleri-Broët

**Affiliations:** 1 INSERM U472, Faculté de Médecine, Paris Sud, France; 2 Service d'Oncologie Médicale, Assistance Publique-Hôpitaux de Paris, UFR Paris VI, France; 3 Service Central d’Anatomie Pathologique Hôtel-Dieu, 1, Place du Parvis Notre Dame, Assistance Publique-Hôpitaux de Paris, UFR Paris VI 75181, Paris, France

**Keywords:** ovarian carcinoma, EGFR-1, prognosis

## Abstract

Epidermal growth factor receptor 1 (EGFR-1) overexpression is usually described as linked with a worse prognosis in a variety of tumours of epithelial origin. However, its role in ovarian cancer is still controversial. The aim of the present study was to analyse the prognostic impact of EGFR-1 in a retrospective series of 93 stage III–IV primary ovarian epithelial tumours. All patients, enrolled in a multicentre GINECO prospective clinical trial, were treated with the same platinum-based combination chemotherapy, and were followed up with a median of 69 months. Epidermal growth factor receptor 1 plasma membrane expression, assessed by immunohistochemistry on paraffin-embedded tissues, was correlated with clinical parameters as well as immunohistochemical expression results of HER-2 (c-erbB-2), BAX, BCL-2, p53 and anti-Ki-67, previously studied in the same series of patients. Positive immunostaining for EGFR-1 was seen in 31 of the 93 analysed cases (33%). No correlation was found between EGFR-1 expression and clinical parameters. No correlation was found between EGFR-1 expression and other biological markers, except for HER-2, which was limit for significance. Indeed, among the EGFR-1-negative cases, 10.3% expressed HER-2, whereas the HER-2-expressing tumours accounted for 27.6% of EGFR-1-positive cases (*P*=0.06). Epidermal growth factor receptor 1 overexpression had no prognostic impact on both overall and progression-free survival through univariate and multivariate analyses. The potential effect of EGFR-1 and HER-2 co-expression on targeted therapy against EGFR-1 and/or HER-2 molecules has to be further analysed.

Ovarian cancer is known to have one of the highest mortality rates of all gynaecological malignancies. Due to the lack of reliable tumour markers, most patients present an advanced stage of the disease at the time of diagnosis. Despite a good initial response to first-line chemotherapy, prognosis remains poor due in part to the development of resistance to chemotherapy. According to the International Federation of Gynecology and Obstetrics (FIGO), clinical stage, histological grade and postoperative residual tumour mass are all identified as the most important prognostic factors for survival in patients with ovarian cancers. Clinical factors as well as prognostic models derived from these clinical factors (Clark *et al*, 2001) remain insufficient to predict accurately the outcome for a specific patient. New markers are needed in order to identify groups of patients who may benefit from different therapeutic options. In this paper, we analyse the prognostic role of EGFR-1 immunohistochemical overexpression.

Recently, the interest of the scientific community has increased for receptors with tyrosine kinase activity, since they constitute potential therapeutic targets. Among them are HER-2 and EGFR-1, two of the four known members of the EGFR family. Epidermal growth factor receptor family receptors (ErbB family) play an important role in regulating a wide variety of cellular functions, including regulation of cell cycle, cell death, angiogenesis and cell differentiation (Alper *et al*, 2001). After binding the ligand (epidermal growth factor or transforming growth factor-alpha), EGFR-1 oligomerises with other EGFR-1 molecules or other members of EGFR family (e.g. HER-2). Activation of the intrinsic receptor tyrosine kinase promotes an intracellular pathway, leading to DNA replication and cell division (Carpenter, 1984).

Abnormal expression of EGFR-1 and its ligands has been shown in several human cancers, such as lung (Tateishi *et al*, 1994), gastric (Yonemura *et al*, 1992), oral (Storkel *et al*, 1993; Maurizi *et al*, 1996), breast (Hainsworth *et al*, 1991) and colorectal (Steele *et al*, 1990; Kluftinger *et al*, 1992; De Jong *et al*, 1998) cancers. The overexpression of EGFR-1 in ovarian carcinoma and cell lines derived from this tumour has also been reported. However, the prognostic impact of EGFR-1 expression in ovarian cancers still remains controversial. In a previous work (Camilleri-Broët *et al*, 2003), we showed a poor prognostic impact of HER-2 overexpression, whereas other tested biological markers (Ki-67, p53, BCL-2 and BAX) were not of prognostic significance. In the present study, we analysed EGFR-1 immunohistochemical expression in a series of homogeneous patients with advanced ovarian tumours in order to evaluate its prognostic impact.

## PATIENTS AND METHODS

### Patients

All cases were included in the CEP trial of the GINECO group, which included from February 1994 through June 1997 164 patients with advanced ovarian cancer (range: 18–70 years). All patients had a histological proven epithelial ovarian cancer, stage III or IV according to the FIGO guidelines, a World Health Organisation (WHO) performance status less than 3, no previous chemotherapy and no major organ failure. Chemotherapy regimen consisted of a combination of i.v. cisplatin (75 mg m^−2^), epirubicin (50 mg m^−2^) and cyclophosphamide regimen (CEP) that patients received for six cycles, with a 21-day interval between each cycle. The cyclophosphamide regimen was randomised between a standard dose of 500 mg m^−2^ and a higher dose of 1800 mg m^−2^ with G-CSF support. Following the six-cycle chemotherapy course, patients were regularly monitored at 3 months interval during the first 3 years and every 6 months thereafter. Second-look laparotomy was performed in 114 patients (69.5%).

At the time of our analysis, 134 patients (87%) showed a disease progression, and 117 (71%) deaths have been recorded for the entire population. The median progression-free survival duration was 15 months (95% confidence interval (CI): [13.1–16.5]) and the median overall survival was 32 months (95% CI: [27.7–36.2]). Response to chemotherapy was evaluated either clinically or with a second-look laparotomy, defined as a 50% or greater reduction in the product obtained from the measurement of each lesion and no appearance of new lesions. Response to chemotherapy was observed in 74 patients (88%).

There was no statistical difference in response rate, progression-free survival and overall survival, according to the cyclophosphamide dose in the two treatment arms. For the biological study, we therefore analysed all patients as a homogeneous population. Of the 164 patients included in the clinical trial, slides from 93 tumours obtained from first surgery were available for further immunohistochemical analysis. Clinical and biological variables were not different when comparing the group to the overall population (detailed in Table 1

**Table 1:** Patient characteristics

	**Clinical trial**		**Biological study**	
**Characteristics**	***N*=164**	**%**	***N*=93**	**%**
*Age*				
Median	59		60	
Range	23–74		23–70	
				
*WHO performance status*				
0	41	25.0	22	23.7
1	98	59.8	56	60.2
2	15	9.1	9	9.7
Missing value	10	6.1	6	6.5
				
*FIGO stage*				
IIIa	5	3.0	2	2.2
IIIb	30	18.4	16	17.2
IIIc	90	54.9	55	59.1
IV	34	20.7	20	21.5
Missing value	5	3.0	0	0
				
*Ascitis*				
No	86	52.4	46	49.5
Yes	70	42.7	46	49.5
Missing value	8	4.9	1	1.1
				
*Residual tumour after first laparotomy*				
No residual disease	20	12.2	11	11.8
Residual disease <2 cm	51	31.1	28	30.1
Residual disease >2 cm	89	54.3	54	58.1
Missing value	4	2.4	0	0
				
*Histological type*				
Serous or mixed epithelial	74	45.1	57	61.3
Clear cell	4	2.4	2	2.2
Endometrioid	9	5.5	7	7.5
Undifferentiated	30	18.3	26	28.0
Missing value	47	28.7	1	1.1
				
*Histological grade*				
Grade 1	0	0	0	0
Grade 2	15	9.1	9	9.7
Grade 3	47	28.7	38	40.9
Grade 4	33	20.1	26	28.0
Missing value	69	42.1	20	21.5

). Age was categorised into two groups: (a) ⩽60 years and (b) >60 years.

### Material

For each case, one sample chosen from the first surgery was reviewed, for histological type and grade, by a panel of pathologists unaware of the initial diagnosis (for more details, see our previous work in the same series of patients) (Camilleri-Broët *et al*, 2003).

Out of the 93 cases reviewed, 57 cases (61.3%) were diagnosed as serous or mixed epithelial carcinoma, two (2.2%) were clear cell carcinoma, seven (7.5%) endometrioid and 26 (28.0%) were undifferentiated carcinoma. In non-clear cell carcinoma, a clear cell constituent was found in 10 (10.8%) cases. Among the 93 cases with primary ovarian tumour samples, nine (9.7%) were considered as grade 2, 38 (40.9%) cases as grade 3 and 26 (28%) cases as grade 4 (Table 1).

### Immunohistochemistry

Immunohistochemical study was performed with the monoclonal antibody raised against EGFR-1 (1/10 dilution, NCL-EGFR, clone EGFR.113; Tebu) on slides obtained from fixed and paraffin-embedded tissue. Thin 4 *μ*m slices were deparaffinised in a routine manner, followed by a microwave pretreatment in tampon urea (4 M, pH 7) and 2 h incubation with the primary antibody. A standard streptavidin–biotin–peroxidase method was applied using a commercially available kit (ABCYS Biospa, Milano), including 30 min incubation for each step, and nuclei were counterstained with haematoxylin. The immunohistochemical study was performed in a single laboratory.

All slides were examined by two pathologists without any knowledge of the clinical data, using a double-headed microscope. Epidermal growth factor receptor 1-positive expression was defined as a plasma membrane positivity in more than 10% of tumour cells. Cytoplasmic staining was considered as nonspecific. A highly EGFR-1-expressing squamous cell lung carcinoma was used as a positive control.

### Statistical analysis

The relationship between EGFR-1 status and the categorical variables described just below were tested using the *χ*^2^ test or Fisher's exact test, whichever was appropriate: patient characteristics (age, WHO performance status, FIGO stage, residual tumour volume and ascitis), histological subtype, tumour grade and immunohistochemical results issued from our previous study (Ki-67, BCL-2, BAX, HER-2 expressions).

Overall survival was calculated from the date of surgery to death or last follow-up examination. Progression-free survival was calculated from the date of surgery to progression or last follow-up examination. Survival curves were derived from Kaplan–Meier (Kaplan and Meier, 1958) estimates. Univariate Cox model analysis (Cox, 1972) was performed to estimate and test the prognostic influence of clinical variables and biological markers/immunohistochemical labelling data. Prognostic impact of EGFR-1 overexpression, adjusted for the other prognostic factors, was assessed in multivariate analyses by using the Cox proportional hazards regression model (Cox, 1972) from a backward stepwise selection procedure. In this setting, we have selected for the started model variables associated with prognosis, with a *P*-value less than or equal to 0.20 in univariate analyses. Hazard ratios (HRs) associated with overall survival or progression-free survival are given with their 95% CI. Statistical significance was considered as *P*-values less than 0.05. All these analyses were carried out using the S-Plus software package.

## RESULTS

### Expression of EGFR-1 according to clinical and other biological variables

**Figure 1:**
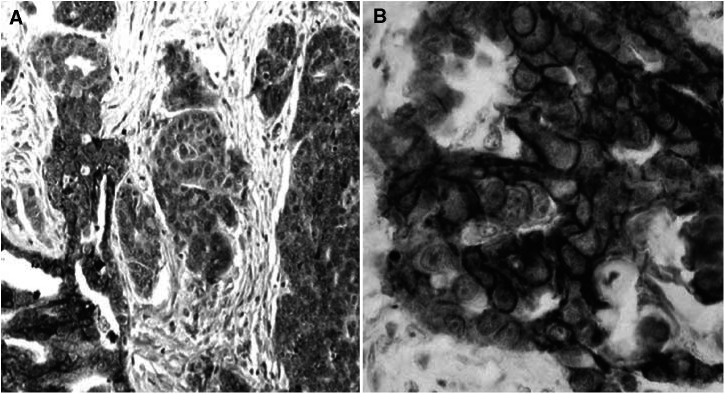
Immunohistochemical labelling results with anti-EGFR-1 antibody-1. (**A**) Heterogeneous plasma membrane expression for EGFR-1 in tumour cells. (**B**) Strong and homogeneous plasma membrane expression for EGFR-1 in tumour cells.

Epidermal growth factor receptor 1 membrane expression has been found in more than 10% of tumour cells in 31 out of the 93 cases tested (33.3%). As we observed previously for HER-2 overexpression in ovarian carcinomas, some cases showed an important heterogeneity of expression (Figure 1

).

No relationship was found between EGFR-1 expression and all tested clinical parameters, as well as the previously studied biological parameters: p53, BAX, BCL-2 and the rate of Ki-67 positivity (Table 2

**Table 2:** Univariate analysis for overall survival and progression-free survival of clinical, histopathological and immunohistochemical parameters

	**Overall survival**		**Progression free survival**	
**Prognostic factor**	**HR [95% CI]**	***P*-values**	**HR [95% CI]**	***P*-values**
*EGFR-1 (% of tumour cells)*	0.83	0.45	0.94	0.81
⩾10% *vs* <10%	[0.51; 1.35]		[0.60; 1.50]	
*Age*	1.07	0.76	1.31	0.23
⩾60 years *vs* <60 years	[0.68; 1.70]		[0.84; 2.03]	
*Performance status*	1.95	0.03	1.50	0.15
1/2 *vs* 0	[1.08:3.52]		[0.87; 2.59]	
*Stage*	1.48	0.19	1.10	0.73
IIIc/IV *vs* IIIa/IIIb	[0.83; 2.66]		[0.63; 1.91]	
*Ascitis*	1.69	0.03	1.71	0.02
Presence *vs* absence	[1.06; 2.69]		[1.09; 2.68]	
*Residual tumour after first laparotomy*	1.15	0.55	1.18	0.46
>2 *vs* <2 cm	[0.73; 1.83]		[0.76; 1.85]	
*Tumour type*	0.81	0.57	1.09	0.79
Clear cell component *vs* others	[0.39; 1.69]		[0.58; 2.07]	
*Tumour grade*	0.92	0.75	0.90	0.70
3/4 *vs* 1/2	[0.54; 1.57]		[0.54; 1.52]	
*Ki-67 expression (% of nuclei surface)*	0.96	0.89	0.81	0.41
⩾30 *vs* <30	[0.56; 1.65]		[0.48; 1.35]	
*BCL-2 (% of tumour cells)*	1.37	0.20	1.19	0.09
⩾10 *vs* <10%	[0.85; 2.21]		[0.94; 2.37]	
*BAX (% of tumour cells)*	0.82	0.41	1.08	0.76
⩾10 *vs* <10%	[0.51; 1.32]		[0.68; 1.71]	
*p53 (% of tumour cells)*	1.11	0.68	1.18	0.49
⩾10 *vs* <10%	[0.69; 1.79]		[0.74; 1.87]	
*HER-2 (% of tumour cells)*	1.99	0.03	2.37	0.01
⩾10 *vs* <10%	[1.08; 3.69]		[1.26; 4.46]	

HR=hazard ratio, CI=confidence interval.

). Among the 87 patients who were tested for both EGFR-1 and HER-2 expression, eight cases (8.6%) co-expressed EGFR-1 and HER-2. Among the EGFR-1-negative cases, six out of 58 (10.3%) expressed HER-2, whereas the HER-2-expressing tumours accounted for eight of the 29 EGFR-1-positive cases (27.6%). This link between EGFR-1 and HER-2 overexpressions was of limited significance (*P*=0.06).

### Patient's outcome and response to chemotherapy

Response to chemotherapy was observed in 50 patients of the 59 EGFR-1-negative patients (85%) and in 24 of the 25 EGFR-1-positive patients (96%). This difference was not significant (*P*=0.27).

At the time of our analysis, out of the 93 patients included in our retrospective series, 80 patients showed a disease progression (86.0%) and 74 patients died (79.6%). The median follow-up was 69 months (95% CI: [58.8–79.0]). Within EGFR-1-overexpressing tumours (31 cases), 28 patients (90.3%) had shown a disease progression, while 24 (77.4%) died.

**Figure 2:**
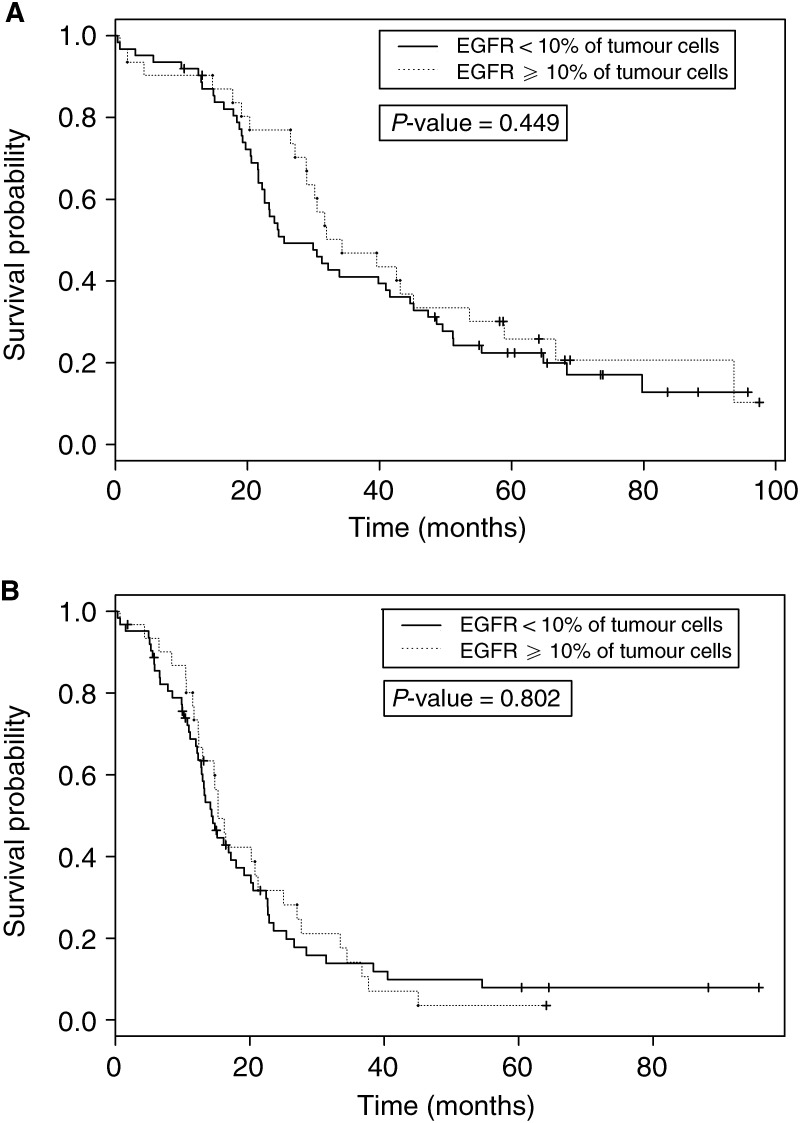
Overall survival (**A**) and progression-free survival (**B**) according to EGFR-1 plasma membrane expression.

In univariate analysis, EGFR-1 expression had no prognostic impact on progression-free survival (*P*=0.80). The median progression-free survival time was 14.3 months (95% CI: [12.9–20.2]) in tumours with EGFR-1-negative cases, whereas it was 15.3 months (95% CI: [13.0–27.0]) in EGFR-1-positive cases (Figure 2A

). In contrast, the presence of an ascitis (*P*=0.02) and HER-2 overexpression by tumour cells (*P*=0.01) were significantly associated with shorter progression-free interval.

When focusing on overall survival, EGFR-1 expression still had no significant prognostic impact (*P*=0.45). The median overall survival duration was 25.6 months (95% CI: [22.6–44.6]) in EGFR-1 negative cases, whereas it was 34.3 months (95% CI: [29.0–38.9]) in EGFR-1-positive cases (Figure 2B). The presence of an ascitis (*P*=0.03), a high performance status (*P*=0.03) and HER-2 overexpression (*P*=0.03) were significantly associated with shorter overall survival.

In the final Cox model for multivariate analysis (Table 3

**Table 3:** Multivariate analysis for overall survival and progression-free survival of FIGO stage, ascitis and HER-2 overexpression

	**Overall survival**			**Progression-free survival**		
**Prognostic factor**	**HR**	**95% CI**	***P*-value**	**HR**	**95% CI**	***P*-value**
*FIGO stage*						
IIIa/IIIb	1					
IIIc/IIId	2.13	1.02–4.42	0.043	—	—	—
						
*Ascitis*						
Absence	1			1		
Presence	1.77	1.06–2.97	0.030	1.96	1.20–3.20	0.008
						
*HER-2*						
<10% of tumour cells	1	1			1	
⩾10% of tumour cells	2.66	1.30–5.44	0.008	3.28	1.61–6.70	0.001

HR=hazard ratio, CI=confidence interval.

), presence of ascitis (*P*=0.008) and HER-2 overexpression (*P*=0.001) both retained a poor prognostic impact on progression-free survival. Regarding overall survival, FIGO stage (*P*=0.043), presence of ascitis (*P*=0.030) and HER-2 overexpression (*P*=0.008) were linked with a poor overall survival. When including EGFR-1 expression variable in the previous multivariate Cox models, it still did not show any prognostic impact (data not shown).

## DISCUSSION

Epidermal growth factor receptor 1 overexpression is thought to be linked with a poor prognosis in several common solid tumours. However, regarding ovarian carcinomas, its prognostic value still remains debated in the literature, showing for some studies a poor prognosis impact (Scambia *et al*, 1992, 1995; Fischer-colbrie *et al*, 1997; Skirnisdottir *et al*, 2001), whereas others showed no influence of EGFR-1 overexpression on patient's outcome (Van der Burg *et al*, 1993; Meden *et al*, 1995; Bartlett *et al*, 1996; Baekelandt *et al*, 1999). Only one study in the literature (Bauknecht *et al*, 1988) showed a favourable prognostic role of EGFR-1 overexpression, but no multivariate analysis was presented. In this series, we have found no significant prognostic impact on the survival time for EGFR-1 overexpression in a homogeneous series of advanced ovarian cancer patients.

These discrepancies in the prognostic role of EGFR-1 may be related to different technical methods used, different antibodies tested, different cutoff values considered, major differences in the rate of EGFR-1-positive cases ranging between 13 and 82%. The advantage of the immunohistochemical technique is to select specifically positive plasma membrane expression of receptors by tumour cells. In contrast, ligand-binding assays give a continuous result, in part dependent on the proportion of tumour cells present in the tissue tested, and requires determining a threshold for positivity.

Four main studies have shown a worse prognosis of the EGFR-1-positive ovarian carcinomas (Scambia *et al*, 1992, 1995; Fischer-colbrie *et al*, 1997; Skirnisdottir *et al*, 2001). The first study of Scambia *et al* (1992) concerned 72 subjects with stage III and IV tumours, whereas the second (1995) concerned 117 patients with all clinical stages. In both studies, the EGFR-1 overexpression status, assayed by a radioreceptor method, led to a rate of 54% positive cases. The EGFR-1 overexpression was not linked to clinical and biological characteristics, but was assessed as an independent prognosis factor. The study of Fischer-Colbrie *et al* (1997) included 108 patients of all clinical stages, with a high rate of positive patients for EGFR-1 overexpression (61%), determined with a radioligand-binding assay. The authors found a poor prognosis impact for EGFR-1 overexpression on progression-free and overall survival in univariate analysis, but no adjustment on clinical variables was made, especially as the part of EGFR-1-positive cases increased significantly with clinical stage in this study. However, these papers, showing a poor prognostic impact of EGFR-1 overexpression, had a higher rate of positive cases, suggesting that the studied positive group may be very different from that of our series. Moreover, two of them analysed a heterogeneous series of patients including tumour stages I–IV. Since EGFR-1 overexpression may increase with the FIGO stage (Fischer-Colbrie *et al*, 1997), the exact impact of such correlation on survival results remains to be investigated. The last study (Skirnisdottir *et al*, 2001) included only early stage (FIGO I and II) epithelial ovarian carcinomas, using thus a population very different from ours.

Among the main papers which showed no prognostic influence of EGFR-1 status (Van der Burg *et al*, 1993; Meden *et al*, 1995; Bartlett *et al*, 1996; Baekelandt *et al*, 1999), two included a large number of patients. Baekelandt *et al* (1999), included 185 homogeneous patients with stage III ovarian cancers. Epidermal growth factor receptor 1-positive immunostaining, observed in 22% of the cases, had a tendency towards worse prognosis in univariate analysis, but was not found to be an independent prognosis factor in multivariate analysis. The study published by Meden *et al* included 266 primary ovarian cancer specimens with FIGO stage I–IV. In all, 13% of the cases were scored positive for EGFR-1 with an immunohistochemical method, showing no significant correlation between EGFR-1 status and overall survival. Our series confirms these findings and is the first study to analyse a series of homogeneous patients (FIGO stage III–IV), included in a multicentre clinical trial followed with a long follow-up (median 69 months).

Epidermal growth factor receptor 1 is one of the promising targets for innovative cancer therapies. Among them, the clinical interest of inhibitors targeting the tyrosine kinase domains of EGFR family (Baselga *et al*, 2002) (e.g. ZD1839, Iressa®, Astra-Zeneca) are under clinical investigation. In contrast to HER-2, whose overexpression predicts clinical response to trastuzumab (Herceptin®), it seems that EGFR-1 overexpression is not necessary for using the therapies targeting its activity (Arteaga, 2002). Indeed, in addition to high expression of the receptor, EGFR-1 signalling may be upregulated by increased expression of its ligands, activating receptor mutations, heterodimerisation with other members of the family such as HER-2, and alterations of downstream molecules in the signalling pathway. Only assessment of these different steps may provide a more complete information on the activation of the EGFR pathway and thus on potential response to EGFR-targeted agents.

Some papers suggest that EGFR-1 overexpression could potentialise the antineoplastic activity of other classical drugs of chemotherapy or ionising radiation *in vitro* or *in vivo* in nude mice (Ciardiello *et al*, 1999; Bianco *et al*, 2002; Kari *et al*, 2003). The principle of such cooperation seems to induce apoptosis, following a downregulation of the antiapoptotic BCL-2 and BCL-XL proteins. In our study, we have found no correlation between BCL-2 and EGFR-1 expressions. In a clinical setting, conflicting results have been reported with regard to response to chemotherapy and EGFR-1 overexpression. In contrast to Scambia *et al*. (1995), who found that EGFR-1 overexpression was of borderline significance in response to chemotherapy, we did not observe such a correlation in our series, similar to that observed by other papers (Baekelandt *et al*, 1999; Ferrandina *et al*, 2002). However, further studies are needed to analyse its potential impact on patients treated with other regimens, including paclitaxel.

In our series, a positive correlation of limited significance (*P*=0.06) was found between EGFR-1 and HER-2 overexpressions, similar to that found by Ferrandina *et al*. (2002) and Skirnisdottir *et al*. (2001). Heterodimerisation of EGFR-1 and HER-2 is functionally active. We have not observed prognostic impact for EGFR-1 and HER-2 co-expressing tumours as compared to the HER-2-positive/EGFR-1-negative group, but this comparison was made with a limited number of patients (data not shown). A recent study from Christensen *et al*. (2001) showed *in vitro* and *in vivo* in nude mice that it is more difficult to inhibit EGFR-1 phosphorylation in cells that express high levels of HER-2 expression. However, in clinical practice, no relationship between HER-2 overexpression and response to ZD1839 (Iressa®) was shown in a series of patients suffering from non-small-cell lung cancer (Cappuzzo *et al*, 2003). Further studies are needed to answer this question in ovarian carcinomas.

In conclusion, our study showed the lack of prognostic impact of EGFR-1 overexpression in a large homogeneous population of stage III and IV ovarian cancer, included in a multicentre clinical trial. However, the absence of prognostic impact eliminates neither a possible role of EGFR-1 in oncogenesis nor a potential benefit of EGFR-targeted agent. Moreover, since a positive correlation of limited significance between EGFR-1 and HER-2 expressions was found, further studies are needed to investigate the clinical effect of such co-expression in a population treated by other regimens, including paclitaxel or targeted therapies.

## References

[bib1] Alper Ö, Bergmann-Leitner ES, Bennet TA, Hacker NF, Stromberg K, Stetler-Stevenson WG (2001) Epidermal growth factor receptor signaling and the invasive phenotype of ovarian carcinoma cells. J Natl Cancer Inst 93: 1375–1384 11562388 10.1093/jnci/93.18.1375

[bib2] Arteaga CL (2002) Epidermal growth factor receptor dependence in human tumors: more than just expression? Oncologist 7(Suppl 4): 31–39 12202786 10.1634/theoncologist.7-suppl_4-31

[bib3] Baekelandt M, Kristensen GB, Trope CG, Nesland JM, Holm R (1999) Epidermal growth factor receptor expression has no independent prognostic significance in advanced ovarian cancer. Anticancer Res 19: 4469–4474 10650794

[bib4] Bartlett JM, Langdon SP, Simpson BJ, Stewart M, Katsaros D, Sismondi P, Love S, Scott WN, Williams AR, Lessells AM, Macleod KG, Smyth JF, Miller WR (1996) The prognostic value of epiodermal growth factor receptor mRNA expression in primary ovarian cancer. Br J Cancer 73: 301–306 8562334 10.1038/bjc.1996.53 PMC2074444

[bib5] Baselga J, Rischin D, Ranson M, Calvert H, Raymond E, Kieback DG, Kaye SB, Gianni L, Harris A, Bjork T, Averbuch SD, Feyereislova A, Swaisland H, Rojo F, Albanell J (2002) Phase I safety, pharmacokinetic, and pharmacodynamic trial of ZD1839, a selective oral epidermal growth factor receptor tyrosine kinase inhibitor, in patients with five selected solid tumor types. J Clin Oncol 20: 4292–4302 12409327 10.1200/JCO.2002.03.100

[bib6] Bauknecht T, Runge M, Schwall M, Pfleiderer A (1988) Occurrence of epidermal growth factor receptors in human adnexal tumors and their prognostic value in advanced ovarian carcinomas. Gynecol Oncol 29: 147–157 3338667 10.1016/0090-8258(88)90209-0

[bib7] Bianco C, Tortora G, Bianco R, Caputo R, Veneziani BM, Caputo R, Damiano V, Troiani T, Fontanini G, Raben D, Pepe S, Bianco AR, Ciardiello F (2002) Enhancement of antitumor activity of ionizing radiation by combined treatment with the selective epidermal growth factor receptor-tyrosine kinase inhibitor ZD1839 (Iressa). Clin Cancer Res 8: 3250–3258 12374696

[bib8] Camilleri-Broët S, Hardy-Bessard AC, Le Tourneau A, Paraiso D, Levrel O, Leduc B, Bain B, Orfeuvre H, Audouin J, Pujade-Lauraine E (2003) HER-2 overexpression is an independent marker of poor prognosis of advanced primary ovarian carcinoma: a multicenter study of the GINECO group. Ann Oncol 15: 104–112 10.1093/annonc/mdh021 14679128

[bib9] Cappuzzo F, Gregorc V, Rossi E, Cancellieri A, Magrini E, Paties CT, Ceresoli G, Lombardo L, Bartolini S, Calandri C, de Rosa M, Villa E, Crino L (2003) Gefitinib in pretreated non-small-cell lung cancer (NSCLC): analysis of efficacy and correlation with HER2 and epidermal growth factor receptor expression in locally advanced or metastatic NSCLC. J Clin Oncol 21: 2658–2663 12860941 10.1200/JCO.2003.01.039

[bib10] Carpenter G (1984) Properties of the receptor for the epidermal growth factor. Cell 37: 357–358 6327062 10.1016/0092-8674(84)90365-9

[bib11] Christensen JG, Schreck RE, Chan E, Wang X, Yang C, Liu L, Cui J, Sun L, Wei J, Cherrington JM, Mendel DB (2001) High levels of HER-2 expression alter the ability of epidermal growth factor receptor (EGFR) family tyrosine kinase inhibitors to inhibit EGFR phosphorylation *in vivo*. Clin Cancer Res 7: 4230–4238 11751524

[bib12] Ciardiello F, Bianco R, Damiano V, De Lorenzo S, Pepe S, De Placido S, Fan Z, Mendelsohn J, Bianco AR, Tortora G (1999) Antitumor activity of sequential treatment with topotecan and anti-epidermal growth factor receptor monoclonal antibody C225. Clin Cancer Res 5: 909–916 10213228

[bib13] Clark TG, Stewart ME, Altman DG, Gabra H, Smyth JF (2001) A prognostic model for ovarian cancer. Br J Cancer 85: 944–952 11592763 10.1054/bjoc.2001.2030 PMC2375096

[bib14] Cox DR (1972) Regression models and life tables (with discussion). J R Stat Soc B 34: 187–202

[bib15] De Jong KP, Stellema R, Karrenbeld A, Koudstaal J, Gouw AS, Sluiter WJ, Peeters PM, Slooff MJ, De Vries EG (1998) Clinical relevance of transforming growth factor alpha, epidermal growth factor receptor, p53 and Ki67 in colorectal liver metastases and corresponding primary tumors. Hepatology 28: 971–979 9755233 10.1002/hep.510280411

[bib16] Ferrandina G, Ranelletti FO, Lauriola L, Fanfani F, Legge F, Mottolese M, Nicotra MR, Natali PG, Zakut VH, Scambia G (2002) Cyclooxygenase-2 (COX-2), epidermal growth factor receptor (EGFR), and Her-2/neu expression in ovarian cancer. Gynecol Oncol 85: 305–310 11972392 10.1006/gyno.2002.6620

[bib17] Fischer-Colbrie J, Witt A, Heinzl H, Speiser P, Czerwenka K, Sevelda P, Zeillinger R (1997) EGFR and steroid receptors in ovarian carcinoma: comparison with prognostic parameters and outcome of patients. Anticancer Res 17: 613–619 9066588

[bib18] Hainsworth PJ, Henderson MA, Stillwell RG, Bennett RC (1991) Comparison of EGFR, c-erbB-2 product and ras p21 immunohistochemistry as prognostic markers in primary breast cancer. Eur J Surg Oncol 17: 9–15 1671658

[bib19] Kaplan EL, Meier P (1958) Non parametric estimation from incomplete observations. J Am Stat Assoc 53: 457–481

[bib20] Kari C, Chan TO, Rocha de Quadros M, Rodeck U (2003) Targeting the epidermal growth factor receptor in cancer: apoptosis takes center stage. Cancer Res 63: 1–5 12517767

[bib21] Kluftinger AM, Robinson BW, Quenville NF, Finley RJ, Davis NL (1992) Correlation of epidermal growth factor receptor and c-erbB2 oncogene product to known prognostic indicators of colorectal cancer. Surg Oncol 1: 97–105 1285215 10.1016/0960-7404(92)90062-p

[bib22] Maurizi M, Almadori G, Ferrandina G, Distefano M, Romanini ME, Cadoni G, Benedetti-Panici P, Paludetti G, Scambia G, Mancuso S (1996) Prognostic significance of epidermal growth factor receptor in laryngeal squamous cell carcinoma. Br J Cancer 74: 1253–1257 8883413 10.1038/bjc.1996.525 PMC2075924

[bib23] Meden H, Marx D, Raab T, Kron M, Schauer A, Kuhn W (1995) EGF-R and overexpression of the oncogene c-erbB-2 in ovarian cancer: immunohistochemical findings and prognostic value. J Obstet Gynaecol 21: 167–178 10.1111/j.1447-0756.1995.tb01090.x 8556578

[bib24] Scambia G, Benedetti-Panici P, Battaglia F, Ferrandina G, Baiocchi G, Greggi S, De Vincenzo R, Mancuso S (1992) Significance of epidermal growth factor in advanced ovarian cancer. J Clin Oncol 10: 529–535 1548517 10.1200/JCO.1992.10.4.529

[bib25] Scambia G, Benedetti-Panici P, Ferrandina G, Distefano M, Salerno G, Romanini ME, Fagotti A, Mancuso S (1995) Epidermal growth factor, oestrogen and progesterone receptor expression in primary ovarian cancer: correlation with clinical outcome and response to therapy. Br J Cancer 72: 361–366 7640219 10.1038/bjc.1995.339 PMC2033999

[bib26] Skirnisdottir I, Sorbe B, Seidal T (2001) The growth factor receptors HER-2/neu and EGFR, their relationship, and their effects on the prognosis in early stage (FIGO I–II) epithelial ovarian carcinoma. Int J Gynecol Cancer 11: 119–129 11328410 10.1046/j.1525-1438.2001.011002119.x

[bib27] Steele RJ, Kelly P, Ellul B, Eremin O (1990) Epidermal growth factor receptor expression in colorectal cancer. Br J Surg 77: 1352–1354 2276016 10.1002/bjs.1800771211

[bib28] Storkel S, Reichert T, Reiffen KA, Wagner W (1993) EGFR and PCNA expression in oral squamous cell carcinomas – a valuable tool in estimating the patient's prognosis. Eur J Cancer B Oral Oncol 29B: 173–277 11706420 10.1016/0964-1955(93)90047-i

[bib29] Tateishi M, Ishada T, Kohdono S, Hamatake M, Fukuyama Y, Sugimachi K (1994) Prognosic influence of the co-expression of epidermal growth factor receptor and c-erbB-2 protein in human lung adenocarcinoma. Surg Oncol 3: 109–113 7952390 10.1016/0960-7404(94)90006-x

[bib30] Van der Burg ME, Henzen-Logmans SC, Foekens JA, Berns EM, Rodenburg CJ, van Putten WL, Klijn JG (1993) The prognostic value of epidermal growth factor receptors, determined by both immunohistochemistry and ligand binding assays, in primary epithelial ovarian cancer: a pilot study. Eur J Cancer 29A: 1951–1957 8280488 10.1016/0959-8049(93)90451-k

[bib31] Yonemura Y, Takamura H, Ninomiya I, Fushida S, Tsugawa K, Kaji M, Nakai Y, Ohoyama S, Yamaguchi A, Miyazaki I (1992) Interrelationship between transforming growth factor-alpha and epidermal growth factor receptor in advanced gastric cancer. Oncology 49: 157–161 1574253 10.1159/000227031

